# Is authorship by women in Brazilian academic surgery increasing? A five-year retrospective analysis

**DOI:** 10.1371/journal.pgph.0000294

**Published:** 2022-04-27

**Authors:** Mariana Graner, Alexandra M. Buda, Carolina B. Moura, Letícia Campos, Isabella Faria, Paul Truche, Fabio Botelho, Laura Pompermaier, Aline Gil Alves Guilloux, Alexis N. Bowder, Julia Ferreira

**Affiliations:** 1 School of Medicine, University of São Paulo, São Paulo, SP, Brazil; 2 Harvard Medical School—Program in Global Surgery and Social Change, Boston, MA, United States of America; 3 Faculdade de Saúde e Ecologia Humana, Vespasiano, MG, Brazil; 4 Faculty of Medical Sciences Universidade de Pernambuco, Recife, PE, Brazil; 5 Federal University of Minas Gerais, Belo Horizonte, MG, Brazil; 6 Harvey E. Beardmore Division of Pediatric Surgery, Montreal Children’s Hospital, Montreal, Canada; 7 McGill University, Montreal, Canada; The University of British Columbia, CANADA

## Abstract

Women remain underrepresented in 80% of Brazilian surgical specialties, however, women representation within the Brazilian academic surgical literature remains unknown. This study aims to evaluate the gender distribution of first and last authors in Brazilian surgical journals. All publications between 2015 and 2019 from the five Brazilian surgical journals with the highest impact factor were reviewed. The first and last authors’ names were extracted from each article and a predictive algorithm was used to classify the gender of each author. Authors were further classified by surgical field and geographic region to investigate patterns of female authorship among journals, specialties, and region over the study period. Multivariable logistic regression was then used to identify factors independently associated with female authorship. 1844 articles were analyzed; 23% (426/1844) articles had female first authors, and 20% (348/1748) had female last authors. *Acta Cirúrgica Brasileira* was observed to have the highest rates for both first and last female authors (37%, 138/371; 26%, 95/370)) and *Revista Brasileira de Ortopedia* (9%, 48/542; 10%, 54/522) had the lowest rates. Papers with a woman senior author were twice as likely to have a woman first author (OR 1.98, 95% CI 1.51–2.58, p≤0.01). Women’s representation in medicine is increasing in Brazil, yet women remain underrepresented as the first and last authors in the Brazilian surgical literature. Our results highlight the importance of senior women mentorship in academic surgery and demonstrate that promoting female surgeon senior authorship through academic and financial support will positively impact the number of female first authors.

## Introduction

Over the last decade, the world has witnessed continuous growth in the number of graduating women physicians [1]. In 2020, almost 60% of physicians under age 29 in Brazil were women; in 2019, women represented 60% of graduating physicians, and there is already a higher proportion of women than men in pediatrics, obstetrics and gynecology, and internal medicine in Brazil [2]. However, this trend is not reflected among all specialties. In particular, the field of surgery remains male-dominated as only 20% of all surgeons in Brazil are women [3, 4]. Furthermore, gender disparities in academic surgery are widespread, with women holding fewer leadership positions than their male colleagues [5].

As publication rates can be used as indicators of individual academic performance and, therefore, be an important aspect of academic promotion achievement, salary determination, grants, and professional status, it is imperative to understand and address gender inequities within the scientific authorship [6]. Furthermore, a lack of academic support for women who choose careers in academic medicine adds to a potential lack of diversity in the research agenda and service delivery [7]. Moreover, it has been proven that heterogeneous groups outperform homogeneous groups since inclusive environments encourage different perspectives, generating a broader range of ideas and innovations. Diverse groups also perform better in decision-making, are more highly cited, produce more promising health science, besides better-representing society [8–10].

Enhancing the recruitment and optimal contributions of women in academic roles enables the planning and implementation strategies that may positively impact women’s performance on academic, research, medical practice, and leadership levels [7]. Our research has the potential to lay the basis for policy-making related to gender equity in the country’s surgical field by offering a scientific foundation. In addition, this work is one of the first of its kind from a low and middle-income countries (LMICs) and can serve as a model for other LMICs to examine barriers to women’s advancement in academic surgical careers related to authorship.

This study aims to evaluate gender distribution among authors in the top Brazilian surgical journals. We analyzed the distribution of first and last authors over time and assessed whether there are differences according to field-weighted citation index values and surgical specialty.

## Material and methods

### Study design

This retrospective study of authorship by women among Brazilian peer-reviewed surgical literature between 2015 and 2019 investigated the evolution of women as authors during the same period by time-series analysis. This study was conducted and reported following the SAGER guidelines and Strengthening the Reporting of Observational Studies in Epidemiology (STROBE), respectively [11, 12]. The top five Portuguese language surgical journals with the highest impact factors based on the Scimago Journal and Country Rank (SJR) were selected and all published articles within the study period were reviewed. Included journals were *Revista do Colégio Brasileiro de Cirurgiões* (0.48), *Arquivos Brasileiros de Cirurgia Digestiva* (0.56), *Revista Brasileira de Ortopedia* (0.83), *Brazilian Journal of Cardiovascular Surgery* (0.84), and *Acta Cirúrgica Brasileira* (1.01) [13]. All journal articles, published between 2015 and 2019, with at least one author affiliated with a Brazilian institution were included in this study, and data were extracted from Scopus. Duplicate articles and articles related to dentistry were excluded.

PubMed metadata contains the full first names of all authors of a publication. All articles identified through Scopus were downloaded through PubMed, and metadata for each article was extracted into text files. We chose to analyze the first and last authors because these positions are commonly associated with academic promotion [1, 14, 15]. Therefore, first author and last author names were extracted from the PubMed metadata or manually if the article was not indexed in PubMed. For each article, the field-weighted citation index (FWCI) was manually extracted from Scopus, and the subspecialty was manually classified into one of 19 categories by reading the title, keywords, and abstracts. Based on the first author’s institutional affiliation, each article was classified into 27 Brazilian states and five Brazilian regions.

### Gender classification

For this study, we adopted the gender definition established by the Sex and Gender Equity in Research (SAGER) guidelines, in which gender refers to the socially constructed roles, behaviors, and identities of women, men, and gender-diverse people. It influences how people perceive themselves and each other, how they behave and interact, and the distribution of power and resources in society [11, 16]. Genderize.io’s predictive algorithm (https://genderize.io) was used to classify each name as a man’s or woman’s gender [17]. As reported by previous studies, Genderize.io uses an application programming interface to predict someone’s gender based on their given name utilizing a proprietary algorithm. The probability indicates the certainty of the assigned gender, basically the ratio of men to women. They also offer the "count", which is the number of data rows examined to calculate the response [18–20]. A test set of 22 articles was randomly chosen as a sample to compare Genderize.io’s predictive algorithm to the study team assigning gender. The study team performed the test based on the authors’ first names. Genderize.io correctly identified 91% (20/22) of the first names. Therefore, the remaining articles were analyzed using the software. Unknown authors or gender predictions with less than or equal to 90% probability (387 first authors, 413 last authors) were re-classified manually to determine gender based on first name or a Google search for image or pronouns where possible. Names with spelling issues or accents are not eligible to be analyzed by Genderize.io. The 800 authors that were manually classified were mainly classified based on their first names by the study authors, disagreements and doubts were solved by the senior Brazilian surgeon author (JLF).

### Article specialty classification

Before the classification, guidelines were established by a nominal technical group which consisted of one Brazilian female surgeon (JLF), one Brazilian male surgeon (FMBF), one surgical resident (ANB), and one female research fellow in global surgery from the USA, (AB). Three medical students performed the manual classification of specialty using these guidelines. When an article could not be classified by the guidelines, it was submitted independently for both Brazilian surgeons to assign a specialty. Articles were manually classified into 19 categories based on their scope: anesthesiology, basic science, breast surgery, cardiovascular surgery, general surgery, gastrointestinal (GI) surgery, hand surgery, head, and neck surgery, neurosurgery, obstetrics and gynecology, orthopedics/traumatology, otorhinolaryngology, pediatric surgery, plastic surgery, urology, thoracic surgery, vascular surgery, and other. Specialty classification was determined by screening the title, abstract, and full-text articles. The basic science category was used for *in vitro* and *in vivo* animal model experiments. A classification of “other” was used for articles that did not fit into any of the previously mentioned classifications.

### Geospatial mapping

In Brazil, significant variation in the distribution of surgical, anesthesia, and obstetric (SAO) providers exists; therefore, we explored whether this trend was also seen in the distribution of women as first and last authors [21]. SAO providers correspond to all health workers who provide surgical, anesthesia, or obstetric care, irrespective of the level of training or supervision [22]. Data regarding the percentages of women SAO providers per specialty in each state was determined from Ferreira *et al*. [23] utilizing the Demografia [24]. First author affiliation GPS coordinates were determined by manual searches in maps from the *Instituto Brasileiro de Geografia e Estatística* (IBGE [25]) under license CC BY 3.0. Each first author affiliation was mapped using the program QGIS (version 3.14.1-PI). The country and South America shapefiles were obtained from the IBGE repository and Thematic Mapping API [26], respectively. In addition, each article was classified into one of five Brazilian regions (North, Northeast, South, Southeast, Central-West) based on the affiliated institution’s address.

### Statistical analysis

The percentage of articles with a female first or last author was calculated for each journal and each year from 2015 to 2019. For the numerator, we considered the number of articles with a woman first or last author and the denominator as the total number of included articles per journal. We examined the relationship between the gender of the first and last authors. Single-author publications were excluded from this analysis. Chi-squared tests were used to compare categorical variables and t-tests for continuous variables, including proportions of women first and last authors by region. Spearman correlation was used to examine the relationship between the percentage of women SAO providers and the percentage of women first and last authors in each surgical subspecialty. The average FWCI of all female first author publications was compared to the average FWCI of all male first author publications using t-tests. The same was done for the FWCI of female and male last author publications. We used logistic regression to evaluate differences in women authorship rates over time between journals. Statistical significance was set at an alpha value less than 0.05. 95% confidence intervals are reported where appropriate. All analyses were performed using Stata16 (Statacorp LLC; College Station, TX) and R v4.0.3 [27]. The *Bibliometrix* package from R was used to extract data from the text files, and the biblioanalysis tool was used to analyze the articles from the Scopus search [28].

### Ethical considerations for human subject research

All data used and presented in this study were publicly available, and the study does not have any kind of intervention. Therefore, the study was exempt from Institutional Review Board evaluation. This study was conducted and reported following the SAGER guidelines and Strengthening the Reporting of Observational Studies in Epidemiology (STROBE), respectively [11, 12].

## Results

**Fig 1:**
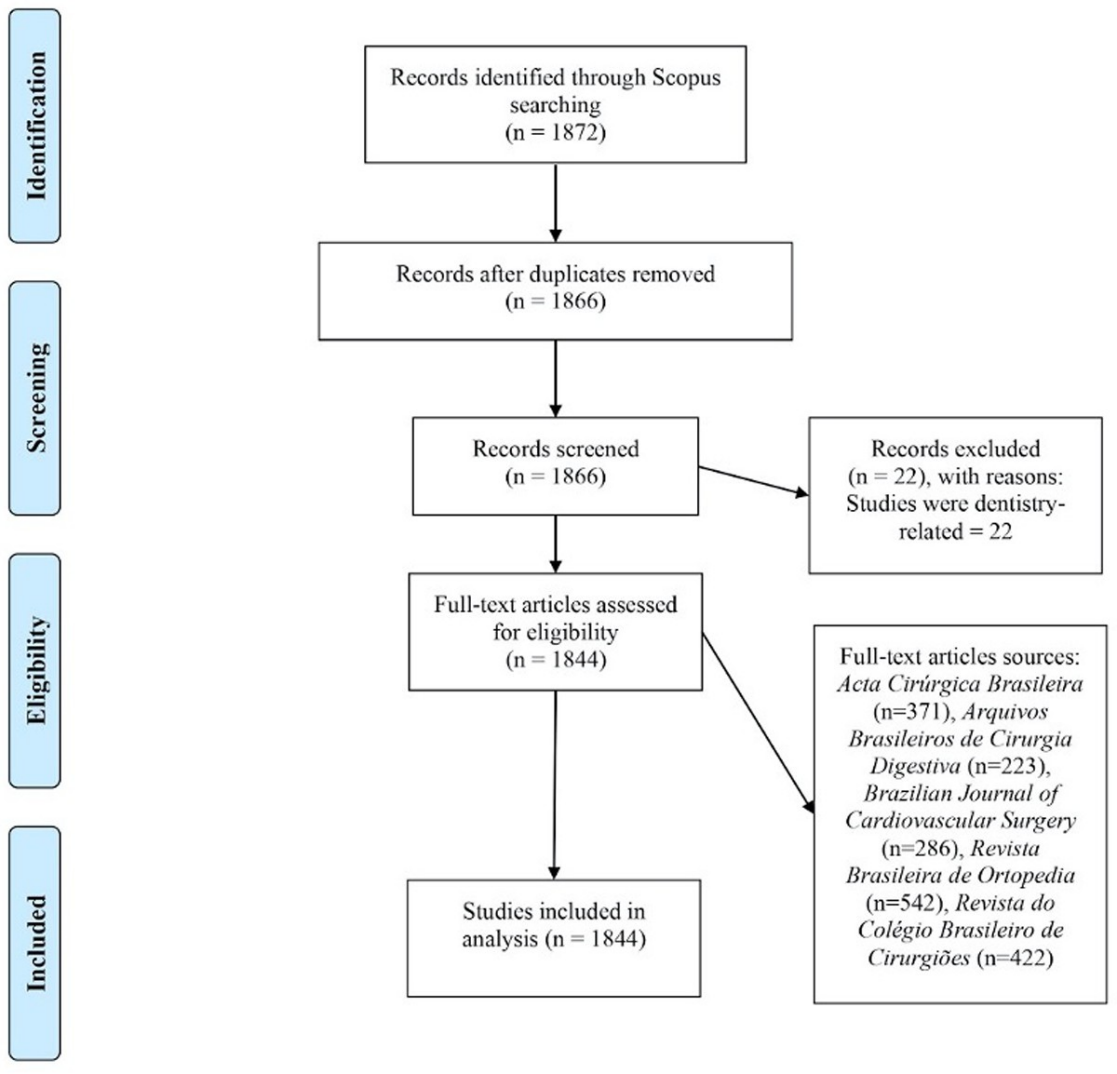
PRISMA journal article selection diagram. This figure depicts the methodology of journal article selection employed in this study, including search strategy, reasons for exclusion, and final record number.

**Fig 2:**
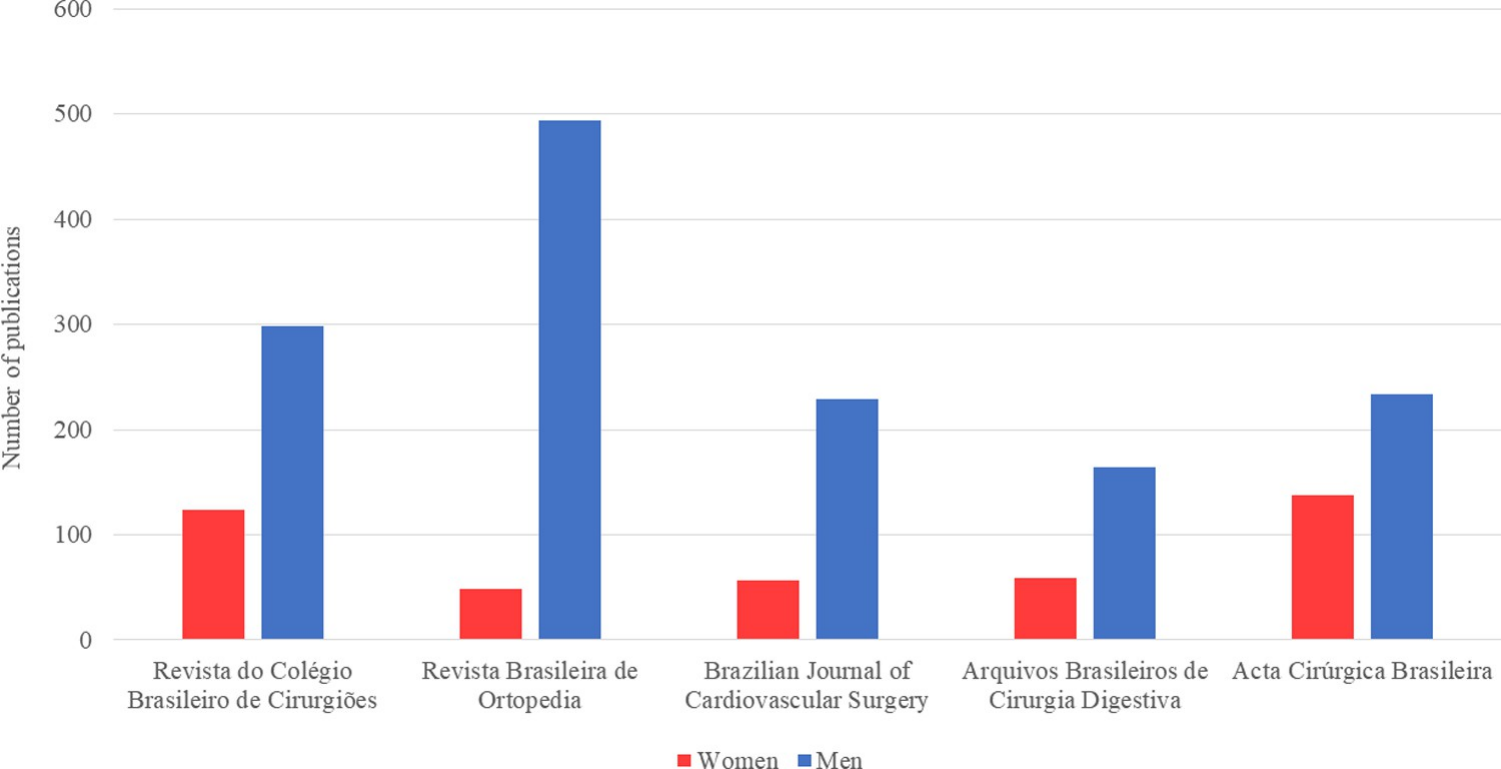
Number of publications by gender for first authors. This figure shows the number of publications for first authors in each journal by gender.

A total of 1872 articles were screened, of which 1844 met inclusion criteria for further analysis (Fig 1). Duplicate articles (n = 6) and dentistry-related articles (n = 22) were excluded. First author and last author names were extracted from PubMed metadata for 1001 articles, while data for the remaining 843 articles were extracted manually (46%). Characteristics of the articles included are reported in Table 1 and Fig 2.

**Table 1:** Percentage of women first and last authors by journal.

Journal	Journal Impact Factor (2017)^a^	Number of Articles	N (%) Women First Author	N (%) Women Last Author	Top 5 Specialties (N articles)	Top 5 First Author Affiliation States (N articles)
Acta Cirúrgica Brasileira	1.008	371	138/371 (37%)	95/370 (26%)	Basic Science (284)	São Paulo (135)
						Paraná (48)
					General Surgery (21)	
						Rio de Janeiro (33)
						Minas Gerais (28)
					GI Tract Surgery (20)	Rio Grande do Sul (22)
					Urology (8)	
					Obstetrics + Gynecology (6)	
Revista do Colégio Brasileiro de Cirurgiões	0.48	422	124/422 (29%)	95/384 (25%)	General Surgery (136)	São Paulo (179)
						Paraná (39)
					GI Tract Surgery (50)	Rio Grande do Sul (34)
					Basic Science (48)	Minas Gerais (32)
					Other (41)	
					Orthopedics + Traumatology (26)	
						Rio de Janeiro (31)
Arquivos Brasileiros de Cirurgia Digestiva	0.561	223	59/223 (26%)	51/217 (24%)	GI Tract Surgery (107)	São Paulo (91)
						Rio Grande do Sul (20)
					General Surgery (81)	Rio de Janeiro (18)
					Basic Science (19)	Paraná (15)
					Other (10)	Minas Gerais (14)
					Pediatric Surgery (2)	
Brazilian Journal of Cardiovascular Surgery	0.842	286	57/286 (20%)	53/255 (21%)	Cardiovascular Surgery (225)	São Paulo (110)
						Minas Gerais (33)
					Other (23)	
						Rio de Janeiro (29)
					Basic Science (20)	
					Vascular Surgery (13)	Paraná (21)
						Rio Grande do Sul (16)
					Anesthesiology (2)	
Revista Brasileira de Ortopedia	0.827	542	48/542 (9%)	54/522 (10%)	Orthopedics + Traumatology (454)	São Paulo (230)
						Rio de Janeiro (55)
					Hand Surgery (38)	
						Paraná (53)
						Minas Gerais (47)
					Basic Science (27)	Rio Grande do Sul (39)
					Other (17)	
					Anesthesiology (1)	

a: Scimago Journal & Country Rank. https://www.scimagojr.com/ (accessed 8 Oct 2020).

**Fig 3:**
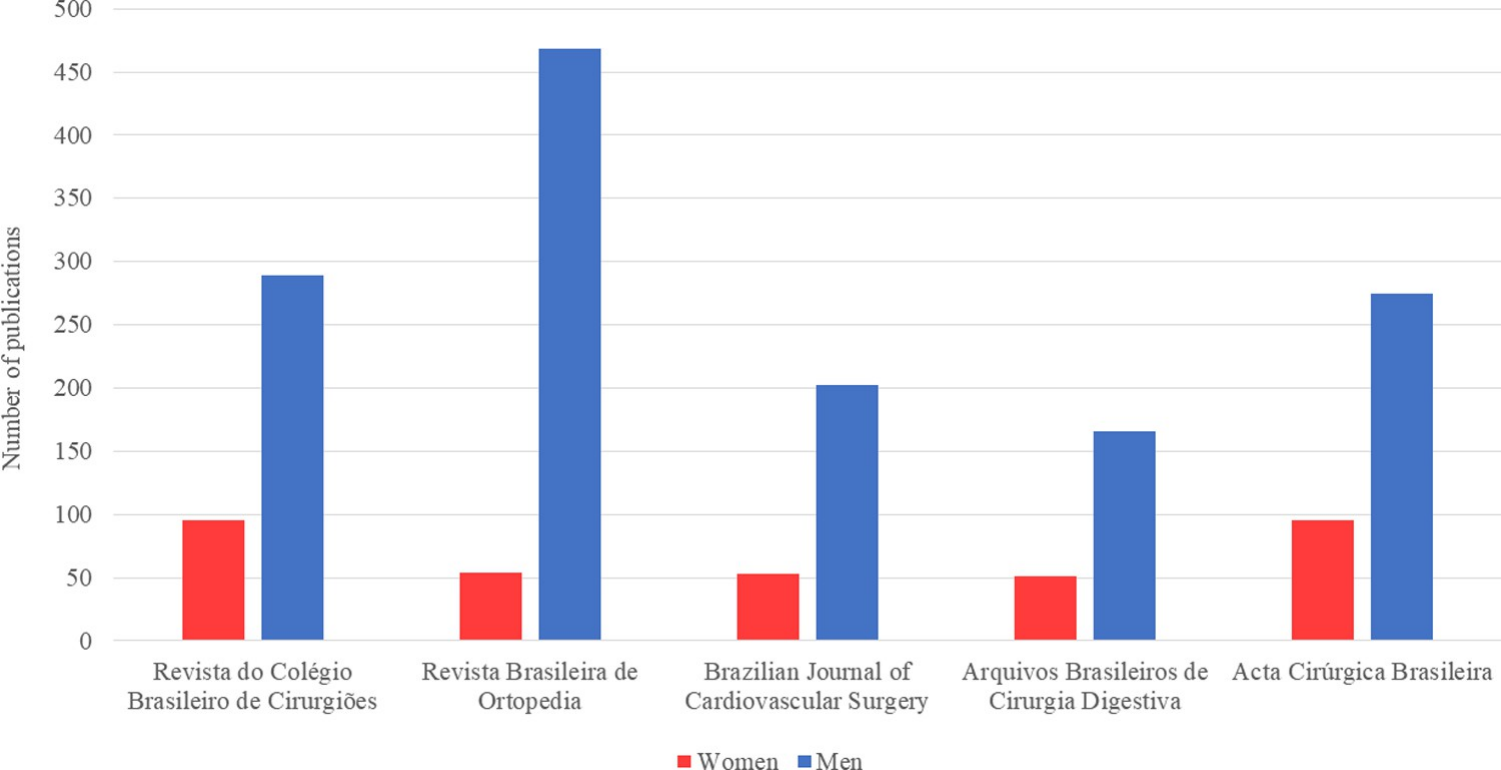
Number of publications by gender for last authors. This figure shows the number of publications for last authors in each journal by gender.

**Fig 4:**
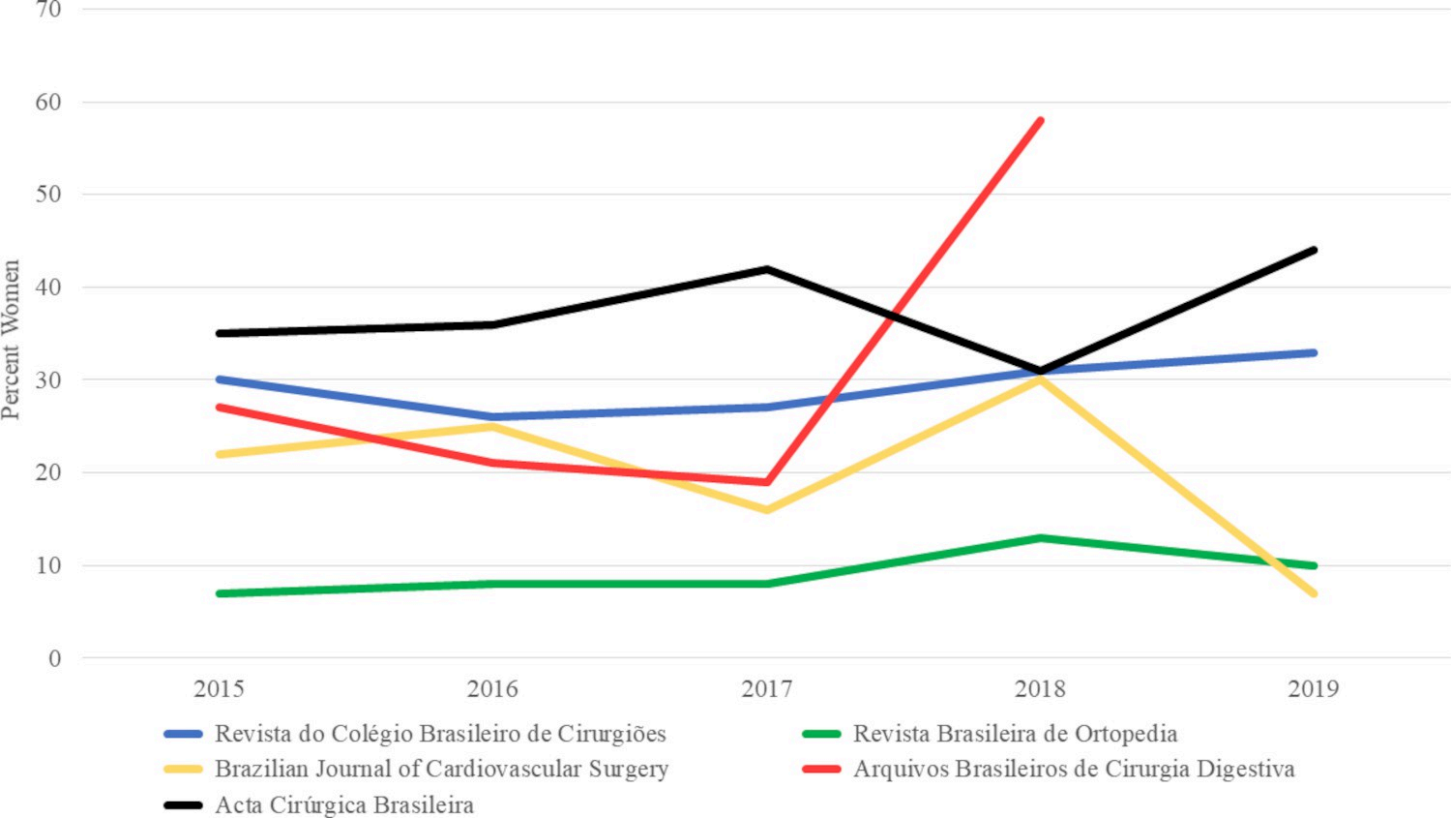
Authorship trends in Brazilian surgical literature 2015–2019 for first authors. This figure demonstrates the changes in authorship trends by the journal over the study period for first authors.

**Fig 5:**
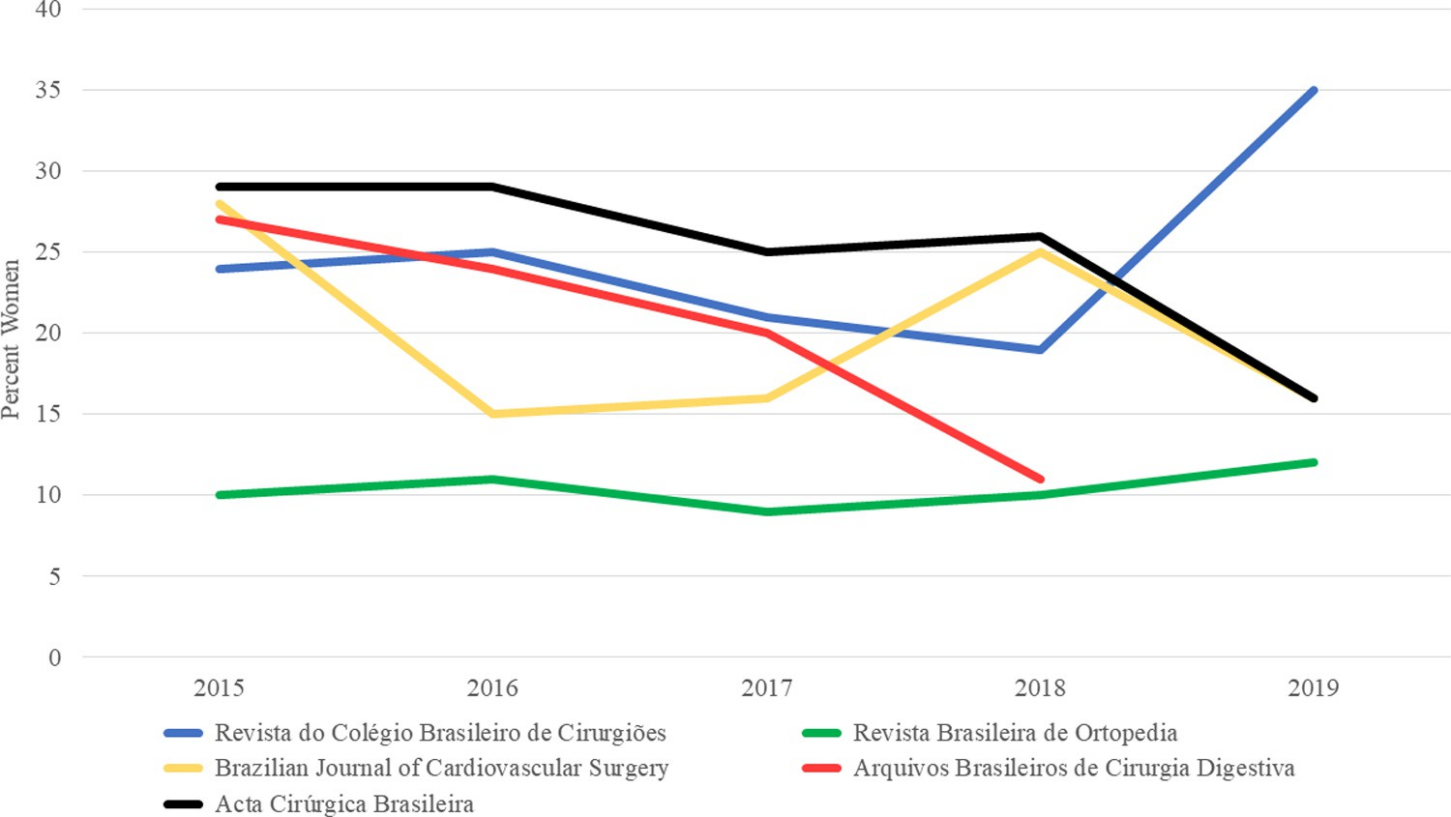
Authorship trends in Brazilian surgical literature 2015–2019 for last authors. This figure demonstrates the changes in authorship trends by the journal over the study period for last authors.

Among the five journals, the percentage of articles with women as first authors ranged from 8 to 58% and from 9 to 35% for the last authors over the 5-year study period (Fig 3). There was a statistically significant difference between the five journals in terms of women’s first authorship (p≤0.001) and last authorship (p≤0.001) (Table 2). From 2015–2019, there was no increase in women’s first authorship [24% (117/496) in 2015 and 22% (57/256) in 2019, p = 0.35], and no increase in the number of articles with a woman as last author. [23% (104/452) in 2015 and 20% (49/245) in 2019, p = 0.25]. Figs 4 and 5 present the trends in Brazilian surgical literature in the last five years for first and last authors by journal, respectively.

**Table 2:** Results of the logistic regression analysis.

	Journals	Odds Ratio	95% Confidence Intervals	P-Value
First Authors^a^	Revista do Colégio Brasileiro de Cirurgiões	0.699	0.519–0.941	0.018
	Revista Brasileira de Ortopedia	0.163	0.113–0.235	<0.001
	Brazilian Journal of Cardiovascular Surgery	0.418	0.292–0.599	<0.001
	Arquivos Brasileiros de Cirurgia Digestiva	0.627	0.433–0.908	0.014
Last Authors^a^	Revista do Colégio Brasileiro de Cirurgiões	0.963	0.693–1.339	0.822
	Revista Brasileira de Ortopedia	0.336	0.233–0.485	<0.001
	Brazilian Journal of Cardiovascular Surgery	0.763	0.521–1.119	0.166
	Arquivos Brasileiros de Cirurgia Digestiva	0.854	0.869–1.038	0.436

^**a**^ Journal was included as a categorical variable in this logistic regression. Acta Cirúrgica Brasileira was the baseline variable because it had the highest percentage of women first and last authors.

Overall, 23% (426/1844) of articles had women first authors, and 20% (348/1748) had women last authors. *Acta Cirúrgica Brasileira* was observed to have the highest rates for both women first and last authors (37%, 138/371; 26%, 95/370), followed by *Revista do Colégio Brasileiro de Cirurgiões* (29%, 124/422; 25%, 95/384), *Arquivos Brasileiros de Cirurgia Digestiva* (26%, 59/223; 24%, 51/217), *Brazilian Journal of Cardiovascular Surgery* (20%, 57/286; 21%, 53/255), and *Revista Brasileira de Ortopedia* (9%, 48/542; 10%, 54/522).

By specialty, the percentage of articles with women first authors ranged from 0–71% (median 19; SD 17) and last authors from 11–33% (median 23; SD 7). Since only a single article from the otolaryngology specialty was identified, this specialty was excluded from the analysis. The highest percentage of articles with women first authors was observed in obstetrics and gynecology (71%, 10/14), followed by basic science (38%, 150/398), and general surgery (33%, 78/239). The lowest percentage of articles with women first authors was found in anesthesiology (0%, 0/9) and neurosurgery (0%, 0/3). Conversely, anesthesiology (33%, 3/9), neurosurgery (33%, 1/3), and other (33%, 16/49) categories had the highest percentage of articles with women last authors, whereas orthopedics/traumatology (11%, 51/480) and hand surgery (11%, 4/37) had the lowest (Table 3).

**Table 3:** Percentage of articles with women first and last authors by subspecialty.

Subspecialty	% women SAO (2020) [24]	% of articles with women first authors	% of articles with women last authors
Anesthesiology	38%	0% (0/9)	33% (3/9)
Basic Science	-	38% (150/398)	27% (105/396)
Breast Surgery	52%	15% (2/13)	23% (3/13)
Cardiovascular Surgery	10%	22% (51/234)	21% (44/211)
General Surgery	22%	33% (78/239)	21% (49/228)
GI Tract Surgery	11%	25% (44/178)	21% (36/171)
Hand Surgery	17%	11% (4/38)	11% (4/37)
Head + Neck Surgery	19%	18% (4/22)	23% (5/22)
Neurosurgery	9%	0% (0/3)	33% (1/3)
Obstetrics + Gynecology	58%	71% (10/14)	29% (4/14)
Orthopedics + Traumatology	7%	8% (37/483)	11% (51/480)
Other	-	27% (26/97)	33% (16/49)
Otolaryngology	41%	100% (1/1)	0% (0/1)
Pediatric Surgery	41%	27% (3/11)	27% (3/11)
Plastic Surgery	24%	23% (6/26)	31% (8/26)
Thoracic Surgery	10%	19% (4/21)	19% (4/21)
Urology	2%	10% (2/21)	14% (3/21)
Vascular Surgery	25%	11% (4/36)	26% (9/35)

We examined whether or not specialties with a higher percentage of women SAO providers had higher percentages of women first and last authors (Table 3). A statistically significant correlation was not found between the rate of women SAO providers by subspecialty and the percentage of women first or last author [p = 0.13, effect size = 0.39, 1-β = 0.45 (% first authors) and p = 0.16, effect size = 0.37, 1-β = 0.41 (% last authors)].

**Fig 6:**
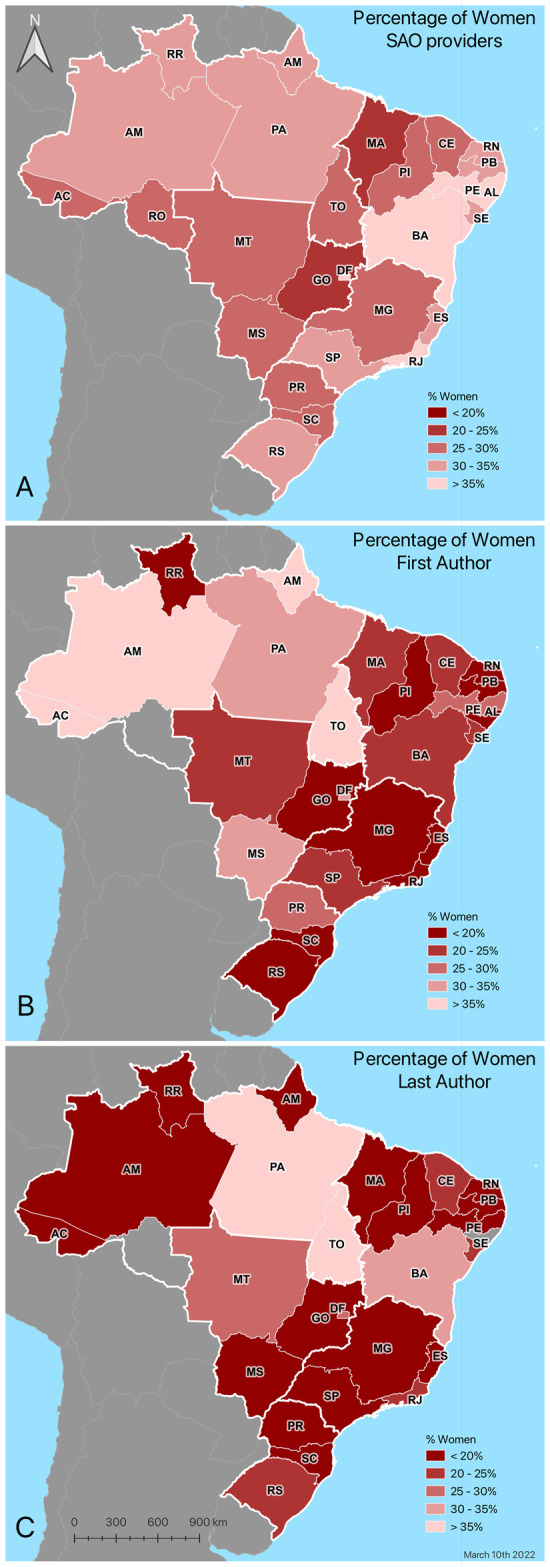
Percent Women SAO Providers (A), Articles with Women First Authors (B), and Articles with Women Last Authors (C) by State. This figure shows the percentage of women SAO providers, percentage of articles with women first authors, and percentage of articles with women last authors by the state in Brazil. Created in QGIS version 3.14.1-PI based on maps from the *Instituto Brasileiro de Geografia e Estatística* (IBGE), CC BY 3.0, on March 10th, 2022. Link to IBGE maps: https://www.ibge.gov.br/en/geosciences/territorial-organization/territorial-meshes/18890-municipal-mesh.html?=&t=o-que-e.

Considering the impact of last author gender on the first author’s gender, 37% (130/348) of women last authors published with women first authors, whereas 20% (283/1400) of men last authors published with women first authors. Women’s last authorship was associated with 1.98 [95% CI 1.51, 2.58 p≤0.01] higher odds of having a woman first author when adjusting for publication year and journal. The percentage of women first and last authors varied by state (Fig 6); however, there was no difference in women first and last authors by region or FWCI. The average FWCI index for women authors was 0.43 [95% CI 0.41, 0.53] compared to the 0.42 [95% CI 0.39, 0.45] average FWCI for men first authors. The average FWCI was 0.42 [95% CI 0.36, 0.48] and 0.44 [95% CI 0.40, 0.47] for women and men last authors respectively.

## Discussion

Although the authorship gender gap in peer-reviewed papers has been described before, this is one of the first studies to explore authorship patterns in Brazil. Among the top five Brazilian surgical journals by impact factor, we found that only one-quarter of academic publications had women first authors, and only one-fifth had women last authors. Despite the increasing presence of Brazilian women in medicine and surgery, the rate of women as first or last authors did not change over the five years studied. This indicates a persistent gender gap in Brazilian surgical academia. Most importantly, we found that women’s last authorship was associated with a higher odds of having a woman first author, highlighting the importance of mentorship by senior female academicians for the growing number of female surgical trainees and new academic surgeons.

International studies reveal gender differences in publication productivity, with women surgeons publishing fewer scientific articles than their men counterparts [29]. Over the last ten years, there has been continuous and slow improvement in authorship by women of high-impact surgical journals [30]. However, these publications capture articles predominantly from North America and Europe, demonstrating a knowledge gap in the other regions of the world [31]. In Global Surgery publications, the biggest gender void is found in authors affiliated with LMICs [32].

The global relevance of Brazil’s gender disparity in surgical authorship publications lies in some of the country’s characteristics. Brazil has a substantial density of physicians, 2.4 physicians per 1,000 people [2], and was one of the first countries to integrate universal health coverage in its legislation, recognizing health as a fundamental right for citizens. Today, the Brazilian health system is a model to the international health community and an example to other countries trying to establish more equitable health systems [33]. In this context, extending the evidence base by including a broader range of countries such as Brazil may improve knowledge about gender bias and challenges women face in academic surgical careers [7]. This could lead to interventions to retain women in surgical careers in Brazil and other LMICs where more surgeons are needed. Women in these countries can be a valuable workforce to provide equitable, safe, and timely surgical care to those who need it; thus, LMICs’ perspectives on gender equity are critical to the global surgery discourse [34].

Previous high-income country studies have shown that women’s first and last authorship were underrepresented in academic publications among surgical subspecialties, such as urology, neurosurgery, and anesthesia [35–37]. Unfortunately, our Brazilian study reaffirmed the same findings. Among surgical subspecialties, we found that obstetrics and gynecology had the highest percentage of women first authors, while the orthopedics journal had the lowest rates of women first and last authors. At first, we thought this might be related to women representing most of Brazil’s obstetric and gynecology workforce [2]. However, we found no association between a higher percentage of women working as surgical, anesthesia, and obstetric providers in a specific subspecialty and higher women authorship in those specialties’ publications, indicating that there are significant barriers for women surgeons publishing in academia.

The effect of gender bias on publications may lead to a lack of women’s authorship in academic surgery. Marrone *et al*. and Filardo *et al*. proposed that any differences in authorship gender proportion that diverge from gender representation in a field may result from bias [15, 31]. Filardo *et al*. named the inherent and unconscious bias where women scientists’ achievements are more likely to be attributed and credited to their men colleagues the “Matilda effect” [31]. Research publications are often associated with academic promotions, leadership roles, and tenure. Therefore, any gender biases that result in a *plateau* or lack of women’s authorship may also prevent women from advancing in surgical academia. Thus, the percentage of women surgeons in a field may not translate into more women as authors or in leadership positions. Many studies have alluded to the lack of women in leadership positions, naming this phenomenon the “leaky pipeline” [7, 10, 38, 39]. The “leaky pipeline” represents the attrition of women in career advancement and appears to be more significant in surgery than in other academic medicine areas [40].

Moreover, women remain underrepresented among the gatekeepers of scientific publishing. A recent study of the top 60 medical journals found that only 16% of editors-in-chief were women, and less than one-fifth of editorial board members were women [41]. As mentioned, this low gender diversity influences not only what gets published but also women’s advancement in the field. This is best illustrated by findings that show journals with women as editors-in-chief have the highest unadjusted rates of women as first authors [31]. Women editors on editorial boards also have been shown to lead to more women being invited to become reviewers [42]. Therefore, having a greater representation of women on editorial boards may represent one strategy to narrow the gender gap in surgical research.

Additional barriers regarding publication for women are centered around research support, dedicated research time, and the publication process itself. Women in academia receive less institutional support and industry sponsorship compared to their men colleagues [43]. Women also receive smaller public and private grants when compared to men in their fields. In addition, differences in academic promotion between men and women can lead to lower academic productivity. This is thought to be because men who often hold more faculty appointments may have more protected time for research [43]. In addition, the increased household, childcare, and childbearing responsibilities of women are other hypotheses as to why some women may have less time to pursue research. Thus, unless positively supported, women who initiate such a career might become discouraged and abandon it [7]. Perhaps, one of the best ways for women who initiate careers to be supported is through woman-to-woman mentorship.

Women’s mentorship may be a key solution for women’s underrepresentation in academic publications among surgical subspecialties. Our study found that women’s last authorship was associated with a higher odds of having a woman first author. This result is mirrored in Bernardi *et al*. and Buda *et al*., who also found a similar correlation between women last authors publishing with women first authors [1, 43]. Mentors increase research productivity and satisfaction in academic medicine [43, 44]. For women, a strong mentorship program correlates with increased retention of women in academia [44]. Moreover, leadership studies demonstrate that most women in senior positions are aware of the responsibility to contribute to the growth of other women, a principle known as “lift as you climb”. This principle associated with a dynamic culture of mentorship is a crucial strategy for developing the next generation of women surgeons as authors and ensuring that they will not face the same barriers as their predecessors [42]. Consequently, an increased representation of women in academia has several implications at organizational and personal levels. First, a heterogeneous group enables intellectual diversity [8]. This may maximize productivity and widen the scope of research priorities and health practices in institutions. Second, a diverse group of academics consolidates a workplace with diminished gender-based discrimination, decreased rates of mental health issues, and better personal life-work balance [8–10, 45, 46]. Third, such a prominent context increases opportunities for women to progress in the field and help each other through woman-to-woman mentorships. This results in women occupying more leadership positions and presenting higher rates of job satisfaction [8, 45].

Our study highlighted that barriers to academic publications exist for women in Brazil and discussed possible solutions. One solution is the diversification of editorial boards to include more women. Then, women in these editorial positions could consider changing the review process so that articles submitted to journals undergo double-blinded review, eliminating possible conscious or unconscious gender bias. A second solution is the development of more woman-to-woman mentorships worldwide. More women around the world “lift as they climb.” Or continue to mentor and support young women medical students interested in academic surgical careers by providing research mentorship. We also propose that when women get promoted across academic institutions in Brazil and other LMIC’s they advocate for and implement ways to address the barriers to academic productivity for women. These systems may include but are not limited to the flexibility of work shifts, improved parental leave policies, and provision of childcare facilities.

This study is not without limitations. First, we only analyzed individual publications and did not examine author H-index, academic positions, or leadership positions. However, academic promotions are often based on the number of individual publications [47], and the H-index does not consider variations based on a specialty like a field-weighted citation index. Second, Genderize.io software could only classify genders into a binary (woman or man); thus, authors identifying as non-binary were not identified. This limitation has been previously discussed in articles concerning gender patterns and identities [48, 49]. However, Genderize.io was used to determine the gender of the authors based on their first names. We tested the algorithm’s accuracy to Brazilian names’ gender against Brazilian study staff, finding that Genderize.io’s predictive algorithm correctly classified 91% of the names in a random sample of articles. Third, since we only chose to examine first and last authorship, the gender of other authors or co-authors was not analyzed. We decided on these two positions because they are most often assessed for academic advancement [1, 14, 15]. Finally, the journals’ subspecialties chosen in this study were selected by Brazilian surgeons, which might confer a regional bias.

## Conclusions

Although more women are entering medicine in Brazil, this is not reflected in the surgical literature, where the number of female first and last authors have not increased over the past five years. Women’s last authorship was associated with higher odds of having a woman first author, indicating the importance of mentorship in academic surgery. Further research is needed to address this disparity, identify specialty-specific gender authorship patterns, and find ways to increase women’s authorship in Brazilian surgical literature. Lastly, publication rates and authorship patterns can be used as a marker of individual performance and promotion and an indicator of gender equity in academic medicine.
